# Psychological reaction to Covid-19 of Italian patients with IBD

**DOI:** 10.1186/s40359-021-00622-6

**Published:** 2021-08-06

**Authors:** Mariarosaria Savarese, Greta Castellini, Salvatore Leone, Enrica Previtali, Alessandro Armuzzi, Guendalina Graffigna

**Affiliations:** 1 EngageMinds HUB – Consumer, Food and Health Engagement Research Center, Faculty of Agricultural, Food and Environmental Sciences, Università Cattolica del Sacro Cuore, Via Milano, 24, 26100 Cremona, Italy; 2 Associazione AMICI Onlus, Milan, Italy; 3 Fondazione Policlinico Universitario A. Gemelli IRCCS, Rome, Italy

**Keywords:** IBD, Covid-19, Patient engagement, Disease management, Psychological outcomes

## Abstract

**Background:**

Patients with inflammatory bowel disease (IBD) may be particularly vulnerable to the effects of the novel coronavirus (Covid-19) on disease management and psychological status. This study explored psychological reactions to the Covid-19 emergency and IBD disease management in a sample of Italian patients.

**Methods:**

An online questionnaire was designed to assess general concerns, psychological reaction, disease management, socio-demographics, and clinical information with validated scales and ad hoc items. A non-probabilistic purposive sample was selected, comprised patients with IBD who belonged to the Italian Association for patients with IBD (AMICI Onlus) completed the questionnaire in April 2020. Data obtained were analyzed using descriptive statistics, student’s T-test for independent groups, and one-way ANOVA (Analysis of Variance).

**Results:**

One thousand fourteen eligible questionnaires were analyzed. Italian patients with IBD appeared to be very worried about the Covid-19 emergency (60.7%) and concerned about the risks of infection (59%). Half of the sample reported medium to high-perceived stress, and 74% had low-medium coping self-efficacy levels. One third was in a state of psychological arousal. Twenty-nine percent of patients had canceled hospital appointments for fear of contracting the virus. The majority of responders believed that belonging to the Italian Association for Patients with IBD - AMICI Onlus - is useful.

**Conclusions:**

The results revealed that this sample of Italian patients with IBD lived with medium level of stress and with inadequate coping self-efficacy regarding disease management. Accordingly, Covid-19 may affect self-management behaviors. Therefore, national and regional associations for patients with IBD, should largely support these patients in this emergency.

**Supplementary Information:**

The online version contains supplementary material available at 10.1186/s40359-021-00622-6.

## Background

Coronavirus disease 2019 (Covid-19) is a viral respiratory illness caused by the novel coronavirus 2019 (2019-nCoV) originating from bats [1]. Risk factors for severe illness include older age and pre-existing comorbid conditions, such as cardiovascular disease, hypertension, diabetes, chronic respiratory disease, and cancer [2]. According to global statistics, the mortality rate of Covid-19 is 4.3% [3]. Northern Italy registered particularly high fatality rates [4], reaching 13%.

After the first Covid-19 case of secondary-transmission was diagnosed in Italy (February 18, 2020), the Italian Government implemented preventive measures to limit the disease’s spread. On March 9, the *#stayathome* Ministerial decree confined the entire population at home, allowing travel only for work, health, or very urgent situations.

These recommendations had a disruptive effect on people’s lifestyles, requiring all to modify their daily habits. Moreover, these measures—together with the uncertainty of the health emergency—had emotional and psychological consequences, challenging people’s abilities to cope with the stressful situation [5].

People with a chronic disease, such as inflammatory bowel disease (IBD), are particularly vulnerable because they must change their daily habits dramatically and eventually modify their disease management to adapt to the emergency [6]. IBD is a life-long condition affecting around 250,000 Italians [7]. As there is no ultimate cure for IBD, treatments include mainly medications with possible unpleasant side effects and—in some cases—invasive surgery, along with constant lifestyle and diet management [8]. Moreover, it is known that patients with IBD are more likely to experience the psychological burden and stress-related disorders compared to other people [9]. The level of stress negatively influences the course and the severity of the underlying intestinal disease [10, 11] and quality of life [12]. It is known to alter the so-called “brain-gut axis,” [13] a link between the central nervous system and the enteric nervous system, which can be exacerbated by high impact stressful events, such as Covid-19 [5, 14]. In this situation, patients’ psychological abilities to cope with such stressful events are protective factors that help improve outcomes [15, 16]. In addition, the ability to actively manage one’s disease—also defined as Patient Engagement [17, 18]—is considered a fundamental element of effective clinical care processes [19, 20]. To date, only a few studies have reported the first results in this area. Yu et al. [21] published a survey on Chinese patients with IBD, demonstrating no significant effect of restrictions on patients’ disease condition and quality of life within one month after the onset of restrictions. Moreover, few Italian studies have mapped patients’ reactions to the Covid-19 emergency, despite the illness being particularly challenging in this country.

In line with these premises, the present study aimed to explore the psychological reactions of Italian patients with IBD and their concerns about the Covid-19 emergency, with particular attention to possible adverse effects on disease management.

## Methods

### Study design and participants

The survey was conducted using a CAWI (Computer Assisted Web Interviewing) methodology. Data were collected using a questionnaire distributed between April 6 and April 13, 2020, to a purposive sample of patients who belonged to the Italian Association for Patients with IBD – AMICI Onlus. The questionnaire was sent to 4187 patients with IBD who were older than 18 years of age. Overall, 1058 patients completed the questionnaire, with a response rate of 25%. Out of 1058, only 1014 were 100% complete, and these were used in statistical analysis. Moreover, "I prefer not to respond" was added as an optional answer to particularly sensitive general concern questions. If the subject chose this answer, this response was considered as “missing data” to obtain more robust and meaningful results.

### Study measures

The survey used in this study measured the following variables (see Additional file 1: part 1 for the complete survey guide):

*Socio-demographic variables:* a series of socio-demographical data were collected, including: age, sex, level of education, region of residence, urban center size, marital status and income in order to characterize the sample.

*Disease characteristics of the patients*: in particular, two questions regarding the year of diagnosis of their disease and the type of disease (Crohn's disease, Ulcerative colitis, indeterminate colitis) were included in the questionnaire.

*Covid-19 general concerns:* General concerns about Covid-19 risk were assessed with three different ad hoc items that were qualitatively piloted in a small sample of patients to verify their understandability before the national launch of the survey. In particular, participants answered a question regarding how concerned they are about the emergency ranging from 1 (not concerned at all) to 10 (very concerned) and they were also asked to rate from 1 (very little) to 5 (a lot) their perceived risk of being infected by the new Covid-19 virus. Finally, the concern that close people could be at risk of contagion was also measured by one item using a 5-point Likert scale from 1 (very little) to 5 (a lot). Moreover, the perception of Covid-19 emergency as a potential threat in compromising one’s delicate health condition was assessed with four ad hoc items. Three questions were measured by dichotomous, yes/no answers and one was measured using a 5-point likert scale ranging from 1 (“completely disagree”) to 5 (“completely agree”). An example of item is: “Does Covid-19 contribute to the worsening of chronic inflammatory bowel disease?”*Covid-19 testing questions:* For the presence of Covid-19 infection, four questions measured on dichotomous scales were used to explore whether the subjects themselves had undergone the polymerase chain reaction (PCR) test for Covid-19 (yes/no) and what was the result (positive/negative), for themselves and their family. These items were also previously used in other researches [22, 23].

#### The psychological reaction to Covid-19 health emergency

Three validated scales were used as follows:

*Perceived Stress Scale *(*PSS*): Four-item version validated by Cohen et al. was used [24]. The PSS is designed to measure the levels of stress experienced in response to a stressful situation. Higher scores on this scale represent greater stress levels experienced as a result of a stressful situation. All items were assessed on 5-point Likert scales ranging from 0 (“never”) to 4 (“very often”). An example of item is: “In the last month, how often have you felt that you were unable to control the important things in your life?”.

*Coping Self-Efficacy Scale *(*CSE*)*:* This scale was validated by Chesney et al. [25] and contained 13 items grouped into three factors that represent different strategies for coping with specific tasks: (1) Problem-focused composed of 6 items (e.g. ‘break an upsetting problem down into smaller parts’); (2) emotional-focused composed of 4 items (e.g. ‘take your mind off negative thoughts’); (3) relational-focused composed of 3 items (e.g. ‘get emotional support from friends and family’). Higher scores on this scale represent greater perceived confidence (self-efficacy) in performing coping behaviors when faced with life challenges, such as disease management in this case. All items were assessed on 11-point Likert scales ranging from 0 (“cannot do at all”) to 10 (“certain can do”) with a middle point 5 (“moderately certain can do”).

*Patient Health Engagement Sca*le (PHE-s®): A revised version of this measure, developed according to the Patient Health Engagement model [26], assesses the consumers’ health engagement level, defined as “people’s psychological readiness and sense of mastery to become active players in their health management and health risk prevention*.*” Previous studies have demonstrated its robust psychometric properties [27]. This scale contains five ordinal items reflecting the continuum of patient movement across the four levels of the PHE model (Patient Health Engagement model). According to the ordinal nature of the PHE-s®, the median score is a more reliable index to calculate the final subject scores [26]. Based on these scores, each respondent is classified into one of the four stages of health engagement described in the PHE model (i.e., Blackout, Arousal, Adhesion, Eudaimonic Project) [26]. For this study’s purposes, the PHE-s® was slightly revised to adapt the items’ formulation to the specific context of the Covid-19 disease emergency.

#### Disease management during the Covid-19 health emergency

Participants were asked five questions about their disease management during the Covid-19 emergency using a series of dichotomous (yes/no) ad hoc questions. The wording of these ad-hoc questions was first qualitatively piloted with a small sample of patients to verify their understandability. In particular were explored:

- *cancellation of hospital visits*: “Did you cancel hospital visits for a gastrointestinal check-up or treatment for fear of contracting Covid-19?”
- *difficulty in contacting the referring doctor*: “Are you having trouble contacting your doctor because of the Covid-19 emergency?”
- *usefulness of patient associations*: “Are the patient associations of reference for your disease very helpful in supporting you in this time of emergency?”
- *possibility to keep in touch with patient associations:* “Can you stay in touch with patient associations despite the emergency?”
- *management of the use of medicines*: “Did you stop taking your medication because of the spread of Covid-19?”

### Data analysis

Descriptive statistics that included frequencies, percentages, means, and standard deviations were used to analyze the data. After checking the normality of the psychological variable distributions (perception of stress, health engagement, and coping self-efficacy) using the asymmetry and kurtosis values, Student’s *t*-test for independent groups or one-way ANOVA was carried out, as appropriate. Post-hoc analyses were conducted using Bonferroni correction to compare different groups. The continuous age variable was divided into four groups usually used in the research on patients with IBD [28]. Segments were labeled as young (18–30), young-adults (31–45), adults (46–60), and old (> 60). All analyses were carried out with IBM SPSS 20, Armonk, New York.

## Results

### Socio-demographics characteristics of the sample

The sample comprised 1014 Italian patients with IBD from various Italian regions. Overall, 476 (46.9%) patients were male, and 538 (53.1%) were female. They ranged in age from 18 to 84 years (mean age = 48.35 ± 13.20 years), with the majority (27.5%) of them being between 46–55 years old. Most responders completed high school (50.6%), lived in the North-East of Italy (35.1%), lived in urban areas of 10/30,000 inhabitants (21.4%), were married (65.1%), and had at least one child (56.7%). A detailed description of the study sample is given in Table 1.

**Table 1:** Demographic and clinical profile of the sample (n = 1014)

	n	%
**Sex**		
Male	476	46.9
Female	538	53.1
**Age**		
18–25	52	5.1
26–35	131	12.9
36–45	225	22.2
46–55	279	27.5
56–65	232	22.9
> 66	95	9.4
**Education**		
No qualifications	2	0.2
Elementary	5	0.5
Junior high	141	13.9
Senior high	513	50.6
College or university	301	29.7
Master/PhD	52	5.1
**Urban center size**		
Up to 5000 inhabitants	158	15.6
5/10,000 inhabitants	149	14.7
10/30,000 inhabitants	217	21.4
30/100,000 inhabitants	173	17.1
100/500,000 inhabitants	135	13.3
More than 500,000 inhabitants	121	11.9
Missing	61	6.0
**Geographic area**		
North-West	295	29.1
North-East	360	35.1
Center	163	16.1
South and Islands	196	19.8
**Marital status**		
Unmarried	268	26.4
Married/cohabitant	660	65.1
Divorced	72	7.1
Widower/widow	14	1.4
**Children**		
Yes	575	56.7
No	439	43.3
**Net monthly income**		
Up to 600 euro	17	1.7
601–900 euro	27	2.7
901–1200 euro	57	5.6
1201–1500 euro	118	11.6
1501–1800 euro	96	9.5
1801–2500 euro	177	17.5
2501–3500 euro	190	18.7
3501–4500 euro	59	5.8
More than 4500 euro	37	3.6
Missing	236	23.3
**Chronic bowel disease**		
Crohn’s disease	508	50.1
Ulcerative colitis	490	48.3
Unclassified IBD	16	1.6
**Age at diagnosed**		
**< **10	8	0.8
10–19	155	15.3
20–29	334	32.9
30–39	251	24.8
40–49	165	16.3
50–59	78	7.7
> 59	23	2.3

### IBD characteristics of the sample

Most patients had Crohn’s disease (50.1%), 48.3% had ulcerative colitis, and 1.6% had unclassified IBD. Moreover, 64.2% of the subjects had received their IBD diagnosis more than ten years before the survey (Table 1).

### Covid-19 general concerns

The results of the survey assessing general concerns about Covid-19 are presented in Table 2. The participants considered themselves at high-risk for the Covid-19 emergency (risk severity score mean ± SD = 7.9 ± 2.1). Most responders (60.7%) declared they were “very worried” about Covid-19. A large proportion of the participants (59%) believed to be at a high risk of contracting Covid-19. Moreover, most of the participants were deeply concerned that people closest to them might contract Covid-19 (80.7%) because the rapid and extensive spread of this virus seemed to have affected many people close to the participants (36.7%) (Table 2).

**Table 2:** Covid-19 general concerns and contagion risk perception

	n	%	Mean (± SD)
**Risk susceptibility (N = 998)**			3.6 (± 0.90)
Low *(1–2)*	102	10.1	
Medium *(3)*	298	29.4	
High *(4–5)*	598	59.0	
**Risk severity** (N = 983)			7.9 (± 2.1)
Low *(1–3)*	48	4.7	
Medium *(4–7)*	320	31.6	
High *(8–10)*	615	60.7	
**Subject tested for COVID-19 with PCR test (N = 1014)**			
Yes	25	2.5	
No	989	97.5	
**Subject’s result for Covid-19 with PCR test (N = 25)**			
Yes	4	16	
No	16	64	
I do not know	5	20	
**Subject thought to have contracted the virus without testing**			
Yes	61	6.1	
No	578	57.4	
I do not know	368	36.5	
**Risk sensitivity for close contact (N = 636)**			4.1 (± 0.89)
Low *(1–2)*	35	5.5	
Medium *(3)*	88	13.8	
High *(4–5)*	513	80.7	
**Covid-19 diagnosis of close contacts (N = 983)**			
Yes	371	36.7	
No	561	55.5	
I do not know	78	7.7	

The numbers in brackets in italic represent the points of Likert scale that were grouped together

*SD* standard deviation

Only 2.5% of the IBD patients in this sample had been subjected to a PCR test (polymerase chain reaction test) for Covid-19: 16% tested positive for the virus. Interestingly, 37% of the subjects could not even speculate whether they have already had contracted the disease (Table 2).

Importantly, subjects appeared to have little knowledge of the relationship between IBD and Covid-19. Some of them declared that they did not know whether their underlying disease made them more vulnerable to contracting Covid-19 (45.2%) or whether the virus could worsen their IBD (50.1%). On the other hand, most patients were convinced that taking immunosuppressants could increase the probability of contracting Covid-19 (57.8%) and that the stress caused by the pandemic worsened the symptoms of IBD (50.8%) (see Additional file 2: Part 2).

### The psychological reaction to the Covid-19 health emergency

About half of the sample (47.4%) appeared to experience a medium level of stress (1.6 ± 0.69) arising from Covid-19, and 74% had low to medium coping self-efficacy levels (6.2 ± 1.78). Among them, one third applied a focused-problem oriented strategy (35.1%), one third applied an emotional-oriented strategy (30.6%), and about one quarter (23.7%) considered the advice of friends and family to deal with difficult and problematic situations.

Some patients reported being in a psychological adhesion stage and were somewhat ready to be active players in their health management and risk prevention during Covid-19 (55%). A quarter of the participants reported experiencing psychological arousal (25.1%) due to difficulties managing their health (Table 3).

**Table 3:** Psychological description of IBD Italian patients (n = 1014)

	n	%	Mean (± SD)	Md
**Perception of stress**			1.6 (± 0.69)	1.5
Low *(0–1)*	533	52.6		
Medium *(2)*	412	40.6		
High *(3–4)*	69	6.8		
**Coping self-efficacy levels**			6.2 (± 1.78)	6.3
Low *(0–3)*	74	7.3		
Sufficient *(4–7)*	676	66.7		
High *(8–10)*	246	26.0		
*Problem‐focused coping*			6.5 (± 1.82)	6.7
Low *(0–3)*	44	4.3		
Medium *(4–7)*	614	60.6		
High *(8–10)*	356	35.1		
*Emotion‐focused coping*			6.1 (± 2.18)	6.1
Low *(0–3)*	119	11.7		
Medium *(4–7)*	585	57.7		
High *(8–10)*	310	30.6		
*Friends and family‐focused coping*			5.6 (± 2.3)	5.7
Low *(0–3)*	195	19.2		
Medium *(4–7)*	579	57.1		
High *(8–10)*	240	23.7		
**Patient health engagement**			4.9 (± 1.18)	5
Blackout	13	1.3		
Arousal	255	25.1		
Adhesion	558	55.0		
Eudaimonic project	188	18.5		

The numbers in brackets in italics represent the points of the Likert scale that were grouped together

*SD* standard deviation, *Md* median

The perception of stress and the patient health engagement scores were also compared by sex, geographical area of residence, age group, and type of disease (Table 3). Males were more engaged in their health [*t* = 6.823; *p* = 2.0E−11; Cohen's d = 0.43], and they had lower degrees of perceived stress compared to women [*t* = -6.899; *p* = 9E−12; Cohen's d = 0.43]. Regarding the levels of coping self-efficacy, males had higher levels (better coping) than females [*t* = 5.233; *p* = 1.87896E−7; Cohen's d = 0.33] (Table 4).

**Table 4:** Main results from male and female comparison (n = 1014)

	Groups			*p* value
	Male mean score ± SD (n = 476)	Female mean score ± SD (n = 538)	Total mean score ± SD (n = 1014)	
Stress perceived (PSS)	1.43 (± .67)	1.72 (± .68)	1.6 (± .69)	9E−12
Coping self-efficacy levels (CSE)	6.52 (± 1.69)	5.94 (± 1.83)	6.2 (± 1.78)	1.87896E−7
Health engagement levels (PHE)	5.22 (± 1.09)	4.73 (± 1.21)	4.9 (± 1.18)	2.0E−11

Moreover, after applying Bonferroni-correction, setting the *p *value to 0.008 given the six comparisons between four [29] geographic area groups, we observed that these significantly influenced health engagement levels [F_(3,1010)_ = 5.261; *p* = 0.001326, η2 = 0.02]. In particular, people who lived in the north-west and north-east were more engaged in their health compared to those who lived on the islands or the south (Table 5).

**Table 5:** Main results from geographic residence area and age groups comparison (N = 1014)

	Health engagement levels (PHE)	Stress perceived (PSS)
**Geographic residence area**		
North-West (n = 295)	Mean = 5.07; SD = 1.09	
North-East (n = 360)	Mean = 5.04; SD = 1.17	
Center (n = 163)	Mean = 4.90; SD = 1.16	
South And Islands (n = 196)	Mean = 4.69; SD = 1.30	
**Age groups**		
18–30 (n = 113)	Mean = 5.03; SD = 1.19	Mean = 1.75; SD = 1.76
31–45 (n = 295)	Mean = 4.84; SD = 1.21	Mean = 1.63: SD = 1.63
46–60 (n = 420)	Mean = 4.90; SD = 1.16	Mean = 1.58; SD = 1.58
> 60 (n = 186)	Mean = 5.26; SD = 1.11	Mean = 1.44; SD = 1.44

	Geographic residence area comparisons					
	North-West/North-East	North-West/Center	North-West/South And Islands	North-East/Center)	North-East/South And Islands	Center/South And Islands
Health engagement levels (PHE)	.027 (.091)	.175 (.114)	.386 (.107) (*p* = 0.002189)	.147 (.110)	.358 (.104) (*p* = 0.003486)	0.211 (.124)

	Age groups comparisons					
	18–30/31–45	18–30/46–60	18–30/> 60	31–45/46–60	31–45/> 60	46–60/> 60
Stress perceived (PSS)	.127 (.076)	.177 (.072)	.313 (.081) (*p* = 0.000846)	.049 (.065)	.186 (.064)	.137 (.061)
Health engagement levels (PHE)	.185 (.129)	.123 (.124)	− .232 (.139)	− .061 (.089)	− .416 (.109) (*p* = 0.000931)	− .355 (.103) (*p* = 0.003611)

Values in cells are differences in means. Standard errors are reported in brackets

*SD* standard deviation

However, the effects of the geographic area on the perceived stress scores [F_(3,1010)_ = 3.715; *p* = 0.012] and coping self-efficacy levels [F_(3,1010)_ = 1.492; *p* = 0.215] were non-significant. Finally, after applying the Bonferroni-correction, setting the *p *value to 0.008 given the six comparisons between four age groups, we observed that age had a significant effect on the perceived stress scores [F_(3,1010)_ = 5.392; *p* = 0.001104, η2 = 0.02] and on the Health engagement [F_(3,1010)_ = 5.467; *p* = 0.000995]. In particular, older patients (> 60 years) had lower levels of perceived stress compared to younger ones (18–30 years). Regarding the Health engagement the results shows that older patients (> 60 years) are more health engaged than young-adults (31–45 years) and adult patients (46–60 years) (Table 5). On the contrary, coping self-efficacy levels [F_(3,1010)_ = 0.765; *p* = 0.514] did not differ with age.

IBD subtypes did not affect coping self-efficacy [F_(2,1011)_ = 0.898; *p* = 0.408], patient health engagement [F_(2,1011)_ = 0.636; *p* = 0.529], or the perceived stress [F_(2,1011)_ = 1.147; *p* = 0.318] (data not shown).

### Disease management during the Covid-19 health emergency

A large portion (71%) of the patients reported that they had not canceled their medical visits and had continued their medications (96.5%) during the Covid-19 emergency. Furthermore, they had kept in touch with patient support and advocacy organizations (such as the Italian Association for Patients with IBD – Amici Onlus) (76.8%), which they considered very helpful (76.4%). About 62% of the sample had contacted their doctor during the Covid-19 emergency (see Additional file 3: Part 3).

## Discussion

This is study has the advantage to have investigated the psychological reactions of a large sample of patients with IBD to Covid-19 in Italy in the very peak of the Covid-19 diffusion and management.

In terms of concerns, these patients reported higher levels of uncertainty during the Covid-19 emergency regarding the possibility of having contracted the virus. In our sample, a considerable number of patients did not know whether the virus could affect their specific disease condition directly or indirectly. In the case of a stressor, such as Covid-19, patients with chronic, immune-mediated conditions, such as IBD, may feel disoriented and abandoned. Such feelings could worsen subjects’ perception of Covid-19’s effect on their disease and quality of life. Previous studies have indeed demonstrated a correlation between feelings of uncertainty and health-related quality of life [30]. Similar to our study, Mosli et al. [31] also found that many Saudi patients with IBD expressed anxiety due to the uncertainty of the Covid-19 pandemic. Our results could help the healthcare professionals and the entire health organizations identifying the main sources of concerns and adopt consequent actions (such as providing patients with information and helping them track contagions) to guarantee better care outcomes. In line with these considerations, recent studies conducted in Italy proposed guidelines for treating these patients as a part of the Covid-19 disease management [32].

Our results regarding psychological reactions showed that Italian patients with IBD have medium levels of perceived stress, with younger patients and females experiencing higher stress, similar to the observations noted in China [21]. In particular, this medium level of perceived stress revealed by this research is similar to that experienced by IBD patients in different parts of the world during the Covid-19 pandemic [33]. Moreover, these findings are also in line with other studies conducted on health adult people during and before this health emergency [34, 35]. Concerning self-efficacy skills, in our study, the great majority of Italian patients with IBD, particularly females, also had low-medium coping self-efficacy levels regarding specific tasks, such as IBD disease management. This is in line with other studies in which women seemed to have a lower psychological ability to cope with stressful events [36]. As the coping ability has been found to play a protective role in IBD [15, 16], particular attention should be given to women with IBD during the pandemic with support interventions. In addition, a significant portion of Italian patients with IBD appears to rely on an emotion-focused coping strategy, which is not the optimal approach to deal with stressful events because contextual factors (such as the variation of imposed preventive measures) could influence emotions in this stressful period, contributing to mood swings. Even in normal conditions, the emotion-focused coping strategy can prevent patients from developing a functional way to deal with risky situations [37, 38]. Accordingly, patients who rely primarily on emotional coping self-efficacy strategy should be targeted for psychological interventions in highly uncertain emergencies, such as Covid-19.

Our study also explored patients’ readiness to actively participate in their health management and risk prevention—defined in the literature as *Patient Engagement* [17]—during this health emergency and demonstrate key self-management behaviors concerning the Covid-19 emergency. Half of our Italian patients with IBD were in the adhesion stage, having a good psychological elaboration of the emergency and sufficiently following the preventive measures to contain the virus spread [27]. In fact, our results also reported a high level of medication adherence and visit continuity. However, almost one-third of our subjects were in blackout and arousal stages, which create a susceptibility to sudden contextual changes impacting them. Indeed, the blackout and arousal stages describe a patient who is psychologically struggling with the current situation, in the grip of negative emotions, and unable to actively manage their disease condition [27]. Such patients are at risk of losing their bearings and falling into a psychological sense of discouragement. As previous research demonstrated, this psychological elaboration of a patient’s identity has a mediating role in their ability to be an active partner of the healthcare system and their adherence to prescriptions [39]. Therefore, identifying and focusing on those patients in lower stages of patient engagement could improve IBD care during the pandemic and facilitate their adhesion to the medical prescriptions.

Considering the socio-demographic characteristics of the sample the results showed that young-adult (31–45 years) and adult (46–60) patients are less engaged in the management of their illness than the older ones (> 60 years). Moreover, people who lived in the North of Italy (north-west and north-east) were more engaged in the management of their illness (inflammatory bowel disease) in this Covid-19 emergency compared to those who lived on the islands or the south. These results seem to contradict the epidemiological data recorded during the first months of the pandemic. Indeed, the distribution of infected subjects and deaths, was not homogeneous in Italy, respectively about 7 times and 12 times higher in northern than in southern regions [40]. However, in northern Italy, many hospitals have promptly responded to this situation by creating new units or converting others to assist patients with Covid-19, training staff and giving guidelines to citizens to be able to protect themselves from Covid-19 contagion. In particular, some northern hospitals specialized in the treatment of IBD patients (such as Policlinico San Donato Research Hospital located in the southeastern part of the Milan metropolitan area), during the pandemic of Covid-19 have tried to support patients with IBD, providing adequate facilities such as the counseling service which required the presence of at least one person (such as IBD-dedicated nurse) available 24/7 to answer e-mails and phone calls [41]. However, in the south of Italy, already known for having a weaker health system than in the north, there have been greater problems managing this pandemic due to the lack of available beds and staff. This different organization and readiness to cope with the Covid-19 pandemic between the north and south could have affected people's perceptions of effectiveness. In particular, those who lived in the north of Italy could feel effective and able to manage their illness because they were surrounded by a system that was able to reassure them and provide information on how to treat their IBD during the pandemic. On the contrary, those who lived in the south could feel more disoriented and less effective in managing their disease during Covid-19 due to lack of support from the local health care system.

This paper has strengths and limitations. In particular, the main strength of this research is that it aims to investigate the psychological status of patients with IBD during the Covid -19 pandemic, being complementary to other studies aimed at understanding the physical symptoms and diagnostic-therapeutic management of IBD patients with Covid-19 [42–44]. Therefore this paper broadens the study horizons related to IBD patients during the Covid-19 health emergency by introducing results that explore the psychological conditions and outcomes of patients with IBD, which are still little considered in literature.

Regarding to limitations, it has to be noted that 75% of the patients did not respond to the survey. This may be because our survey was implemented during the first month of emergency when the patients were the most likely to be emotionally overcome. Nonetheless, many Italian patients with IBD participated in our survey, indicating their willingness to react to the pandemic by contributing with their experiences to our understanding of the current situation and its effect on their daily care routine. Furthermore, in our study, we only relied on patient-reported outcomes rather than chart reviews or other more objective data. This could be, in some ways, a limitation of the study. However, scholars have recently underlined the importance of giving voice to the patients and understanding their lived disease experience, for example, through patient-reported measures of experiences and outcomes [45]. With patients as key stakeholders in the healthcare sector, interest in evaluating their direct experiences and creating added value has been growing. Thus, our results provide a valuable contribution to achieving this aim in the pandemic situation. Another potential limitation was that the participants were recruited from among patients with a long disease duration belonging to the Italian Association for patients with IBD – Amici Onlus. As such, IBD patients in this study were not novices to their disease and its fallouts and were more likely to have developed sufficient ability to deal with their emotions, even in a stressful pandemic situation, which could have contributed to their lower perceived stress in this study. These participants may also be more prepared to deal with stressful situations, as they are “practiced” patients. Finally, in this study, we considered psychometric significance as an indicator of the effect of Covid-19 on Italian patients with IBD’s disease experience, while the relationship between psychological discomfort and IBD symptomatology was not investigated, representing a limitation. Nevertheless, following the methodological recommendations [46], small yet significant psychometric differences reflect the change in IBD patients' psychological well-being and distress. Future studies should verify the relationships of IBD symptoms and actual long-term IBD with Covid-19 related outcomes and the various psychological parameters and outcomes examined in this study. Future studies should also consider implementing an experiment with a healthy control group to compare these two populations.

## Conclusion

The present study was able to contribute to the literature about IBD patients and Covid-19 pandemic, suggesting that it is important to consider and monitor the psychological status of patients with IBD during the Covid-19 pandemic to prevent a worsening in psychological outcomes which can, in turn, have negative implications on clinical ones. In particular, our results still highlighted an important effect of this pandemic on stress, coping, and engagement capabilities. For this reason, we emphasize the need for future studies to further investigate the psychological reaction of patients with IBD and give indications for support interventions.

## Supplementary Information

**Additional file 1.** Questionnaire of Survey.

**Additional file 2.** Graphs about Covid-19 general concerns.

**Additional file 3.** Graphsabout Disease management during the Covid-19 health emergency.

## Data Availability

Not applicable.

## Abbreviations

IBD: Inflammatory bowel disease
ANOVA: Analysis of Variance
CAWI: Computer Assisted Web Interviewing
PSS: Perceived Stress Scale
CSE: Coping Self-Efficacy Scale
PHE-s®: Patient Health Engagement Scale
PHE model: Patient Health Engagement model
PCR test: Polymerase chain reaction test
SD: Standard deviation

## References

[1] Zhu N, Zhang D, Wang W, Li X, Yang B, Song J (2020). A novel coronavirus from patients with pneumonia in China, 2019. N Engl J Med.

[2] Liu W, Tao ZW, Wang L, Yuan ML, Liu K, Zhou L (2020). Analysis of factors associated with disease outcomes in hospitalized patients with 2019 novel coronavirus disease. Chin Med J (Engl).

[3] Haybar H, Khalil K, Rahim F (2020). Underlying chronic disease and Covid-19 infection: a state-of-the-art review. Jundishapur J Chronic Dis Care.

[4] Khafaie MA, Rahim F (2020). Cross-country comparison of case fatality rates of Covid-19/SARS-COV-2. Osong Public Health Res Perspect.

[5] Mazza C, Ricci E, Biondi S, Colasanti M, Ferracuti S, Napoli C (2020). A nationwide survey of psychological distress among Italian people during the Covid-19 pandemic: immediate psychological responses and associated factors. Int J Environ Res Public Health.

[6] Scaldaferri F, Pugliese D, Privitera G, Onali S, Lopetuso LR, Rizzatti G (2020). Impact of Covid-19 pandemic on the daily management of biotechnological therapy in inflammatory bowel disease patients: the re-organizational response in a high-volume Italian IBD Center. United Eur Gastroenterol J.

[7] AMICI (Onlus). AMICI ONLUS associazione nazionale. https://amiciitalia.eu/node/2344. Accessed 17 July 2020.

[8] Kaplan GG (2015). The global burden of IBD: from 2015 to 2025. Nat Rev Gastroenterol Hepatol.

[9] Graff LA, Walker JR, Bernstein CN (2009). Depression and anxiety in iflammatory bowel disease: a review of comorbidity and management. Inflamm Bowel Dis.

[10] Sgambato D, Miranda A, Ranaldo R, Federico A, Romano M (2017). The role of stress in inflammatory bowel diseases. Curr Pharm Des.

[11] Edman JS, Greeson JM, Roberts RS, Kaufman AB, Abrams DI, Dolor RJ (2017). Perceived stress in patients with common gastrointestinal disorders: associations with quality of life, symptoms and disease management. Explor J Sci Health.

[12] Beatriz P, Guillermo V (2019). P093 stressful events possibly related to the onset of IBD. Am J Gastroenterol.

[13] Bernstein CN (2017). The brain-gut axis and stress in inflammatory bowel disease. Gastroenterol Clin N Am.

[14] Sood S (2020). Psychological effects of the Coronavirus disease-2019 pandemic. Res Humanit Med Educ.

[15] van Erp SJH, Brakenhoff LKMP, Vollmann M, van der Heijde D, Veenendaal RA, Fidder HH (2017). Illness perceptions and outcomes in patients with inflammatory bowel disease: is coping a mediator?. Int J Behav Med.

[16] Alameel T, Mosli M (2019). The missing “c”: Crohn’s, colitis and coping. Saudi J Gastroenterol.

[17] Barello S, Graffigna G, Vegni E (2012). Patient engagement as an emerging challenge for healthcare services: mapping the literature. Nurs Res Pract.

[18] Gruman J, Rovner MH, French ME, Jeffress D, Sofaer S, Shaller D (2010). From patient education to patient engagement: implications for the field of patient education. Patient Educ Couns.

[19] Shah SL, Siegel CA (2015). Increasing patient activation could improve outcomes for patients with inflammatory bowel disease. Inflamm Bowel Dis.

[20] Atreja A, Otobo E, Ramireddy K, Deorocki A (2018). Remote patient monitoring in IBD: current state and future directions. Curr Gastroenterol Rep.

[21] Yu M, Ye Z, Chen Y, Qin T, Kou J, Tian D (2020). Questionnaire assessment helps the self-management of patients with inflammatory bowel disease during the outbreak of Coronavirus Disease 2019. medRxiv.

[22] Graffigna G, Barello S, Savarese M, Palamenghi L, Castellini G, Bonanomi A (2020). Measuring Italian citizens’ engagement in the first wave of the Covid-19 pandemic containment measures: a cross-sectional study. PLoS ONE.

[23] Graffigna G, Palamenghi L, Savarese M, Castellini G, Barello S (2021). Effects of the Covid-19 emergency and national lockdown on Italian citizens’ economic concerns, government trust, and health engagement: evidence from a two-wave panel study. Milbank Q.

[24] Cohen S, Kamarck T, Mermelstein R (1983). A global measure of perceived stress. J Health Soc Behav.

[25] Chesney MA, Neilands TB, Chambers DB, Taylor JM, Folkman S (2006). A validity and reliability study of the coping self-efficacy scale. Br J Health Psychol.

[26] Graffigna G, Barello S, Bonanomi A, Lozza E (2015). Measuring patient engagement: development and psychometric properties of the patient health engagement (PHE) scale. Front Psychol..

[27] Graffigna G, Barello S (2018). Spotlight on the patient health engagement model (PHE model): a psychosocial theory to understand people’s meaningful engagement in their own health care. Patient Prefer Adherence.

[28] Delmondes LM, Nunes MO, Azevedo AR, de Oliveira MM S, Coelho LER, Torres-Neto J da R (2015). Clinical and sociodemographic aspects of inflammatory bowel disease patients. Gastroenterol Res.

[29] Lee S, Lee DK (2018). What is the proper way to apply the multiple comparison test?. Korean J Anesthesiol.

[30] Niv G, Bar Josef S, Ben Bassat O, Avni I, Lictenstein L, Niv Y (2017). Quality of life and uncertainty in Crohn’s disease. Qual Life Res.

[31] Mosli M, Alourfi M, Alamoudi A, Hashim A, Saadah O, Al Sulais E, AlAmeel T, Alharbi O, Bakari S, Meeralam Y, Alshobai S, Alsahafi M, Jawa H QY (2020). A cross-sectional survey on the psychological impact of the Covid-19 pandemic on inflammatory bowel disease patients in Saudi Arabia. Saudi J Gastroenterol.

[32] Danese S, Cecconi M, Spinelli A (2020). Management of IBD during the Covid-19 outbreak: resetting clinical priorities. Nat Rev Gastroenterol Hepatol.

[33] Cheema M, Mitrev N, Hall L, Tiongson M, Ahlenstiel G, Kariyawasam V (2021). Depression, anxiety and stress among patients with inflammatory bowel disease during the Covid-19 pandemic: Australian national survey. BMJ Open Gastroenterol.

[34] Vallejo MA, Vallejo-Slocker L, Fernández-Abascal EG, Mañanes G (2018). Determining factors for stress perception assessed with the Perceived Stress Scale (PSS-4) in Spanish and other European samples. Front Psychol.

[35] Zhao SZ, Wong JYH, Wu Y, Choi EPH, Wang MP, Lam TH (2020). Social distancing compliance under Covid-19 pandemic and mental health impacts: a population-based study. Int J Environ Res Public Health.

[36] Skrastins O, Fletcher PC (2016). “one Flare at a Time”: adaptive and maladaptive behaviors of women coping with inflammatory bowel disease and irritable bowel syndrome. Clin Nurse Spec.

[37] McCauley M, Minsky S, Viswanath K (2013). The H1N1 pandemic: media frames, stigmatization and coping. BMC Public Health.

[38] Austenfeld JL, Stanton AL (2002). Coping through emotional approach: a new look at emotion, coping, and health-related outcomes. J Personal.

[39] Graffigna G, Barello S, Bonanomi A (2017). The role of Patient Health Engagement model (PHE-model) in affecting patient activation and medication adherence: a structural equation model. PLoS ONE..

[40] Di Ciaula A, Palmieri VO, Migliore G, Portincasa P (2020). Covid-19, internists and resilience: the north-south Italy outbreak. Eur J Clin Investig.

[41] Occhipinti V, Pastorelli L (2020). Challenges in the care of IBD patients during the Covid-19 pandemic: report from a “Red Zone” area in northern Italy. Inflamm Bowel Dis.

[42] Singh AK, Jena A, Kumar-M P, Sharma V, Sebastian S (2021). Risk and outcomes of coronavirus disease in patients with inflammatory bowel disease: a systematic review and meta-analysis. United Eur Gastroenterol J.

[43] D’Amico F, Danese S, Peyrin-Biroulet L (2020). Systematic review on inflammatory bowel disease patients with Coronavirus disease 2019: it is time to take stock. Clin Gastroenterol Hepatology.

[44] Ludvigsson JF, Axelrad J, Halfvarson J, Khalili H, Larsson E, Lochhead P (2021). Inflammatory bowel disease and risk of severe Covid-19: a nationwide population-based cohort study in Sweden. United Eur Gastroenterol J.

[45] Bojic D, Bodger K, Travis S (2017). Patient reported outcome measures (PROMs) in inflammatory bowel disease: new data. J Crohn’s Colitis.

[46] Borsboom D, Mellenbergh GJ, Van Heerden J (2004). The concept of validity. Psychol Rev.

