# Supplementary material for: Psychological reaction to Covid-19 of Italian patients with IBD
Source: BMC Psychol. 2021 Aug 6;9:115. doi: 10.1186/s40359-021-00622-6 (PMC8343359; doi:10.1186/s40359-021-00622-6)
Supplement: Supplementary file 1 — Additional file 1. Questionnaire of Survey. [file 40359_2021_622_MOESM1_ESM.docx]

1. **Socio-demographic variables**

**Sex**

- - Male
  - Female

**Year of birth (in numbers)**

|  |
| --- |

**Region of residence**

- - North-West
  - North-East
  - Center
  - South and Islands

**Inhabited centre size**

- - Up to 5.000 inhabitants
  - 5/10.000 inhabitants
  - 10/30.000 inhabitants
  - 30/100.000 inhabitants
  - 100/500.000 inhabitants
  - More than 500.000 inhabitants
  - I don’t know

**Level of education**

- No qualifications
- Elementary
- Junior high
- Senior high
- College or university
- Master/PhD

**Marital status**

- Unmarried
- Married/cohabitant
- Divorced
- Widower/widow

**Do you have childrend?**

- - Yes
  - no

**Net monthly income**

- - Up to 600 euro
  - 601-900 euro
  - 901-1200 euro
  - 1201-1500 euro
  - 1501-1800 euro
  - 1801-2500 euro
  - 2501-3500 euro
  - 3501-4500 euro
  - More than 4500 euro
  - I prefer not to answer

1. **Clinical characteristics of the patients**

***Type of disease***

- Crohn's disease
- Ulcerative colitis
- IBD unclassified

***Year of diagnosis _____________***

1. **Covid-19 general concerns**

***How much are you worried for the COVID-19 emergency? (1=Not at all; 10=A lot)***

*1 2 3 4 5 6 7 8 9 10 I don’t know*

***In particular, how much do you feel at risk of being infected by the new Coronavirus? (1= Not at all 5 = A lot at risk).***

| ***Not at all*** | ***A little*** | ***Nor little nor much*** | ***Quite at risk*** | ***A lot at risk*** |  | ***I don’t have an opinion*** |
| --- | --- | --- | --- | --- | --- | --- |
| ***1*** | ***2*** | ***3*** | ***4*** | ***5*** |  | ***6*** |

***How concerned are you about the risk of someone in your family or network of friends contracting COVID-19 infection? Answer on a scale from 1 to 5 (where 1=not at all worried and 5 = Very worried))***

| ***Not at all worried*** | ***A little worried*** | ***Nor little nor much worried*** | ***Quite worried*** | ***Very worried*** |  | ***I don’t have an opinion*** |
| --- | --- | --- | --- | --- | --- | --- |
| ***1*** | ***2*** | ***3*** | ***4*** | ***5*** |  | ***6*** |

***Have you personally undergone the COVID-19 test?***

- - Yes
  - No

***If yes, did it test positive for COVID-19?***

- Yes
- No
- I don’t know
- I prefer not to answer

***Has anyone of your loved ones (family, friends, colleagues) been diagnosed the new Coronavirus (COVID-19)?***

- Yes
- No
- I don’t know
- I prefer not to answer

***Do you think you have contracted the virus even though you did not do the test?***

- Yes
- No
- I don’t know
- I prefer not to answer

***The spread of Covid-19 can have an impact on MICI management. We ask you to answer the following questions based on your personal experience.***

***Does IBD make those suffering from it more exposed to contract Covid-19?***

- - Yes
  - No
  - I don’t know

***Does Covid-19 contribute to the worsening of IBD?***

- - Yes
  - No
  - I don’t know

***Does taking immunosuppressant drugs make you more exposed to contract Covid-19?***

- - Yes
  - No

***With regard to your pathology, how much do you agree with the following question?***

***From 1=completely disagree to 5=completely agree***

|  | ***Completely disagree*** | ***Disagree*** | ***Nor agree nor disagree*** | ***Agree*** | ***Completely agree*** |
| --- | --- | --- | --- | --- | --- |
| The stress caused by the health emergency worsens the symptoms related to my disease |  |  |  |  |  |

1. **Psychological reaction to Covid-19 health emergency**

***The following questions are about your emotions and thoughts in the last month. For each question, answer indicating how often you feel or think in a certain way (from 0=never to 4=very often)***

|  | **0 = Never** | **1 = Almost never** | **2 = Sometimes** | **3 = Fairly Often** | **4 = Very Often** |
| --- | --- | --- | --- | --- | --- |
| In the last month, how often have you felt that you were unable to control the important things in your life? | O | O | O | O | O |
| In the last month, how often have you felt confident about your ability to handle your personal problems? | O | O | O | O | O |
| In the last month, how often have you felt that things were going your way? | O | O | O | O | O |
| In the last month, how often have you felt difficulties were piling up so high that you could not overcome them? | O | O | O | O | O |

***We ask you to answer the following questions about the emotions you can experience when dealing with difficult situations such as the current Covid-19 emergency.***

***When things are not going well, or when you have problems, how confident do you feel about doing the following things?***

***Answer on a scale from 0 (I can't do it at all) to 10 (I'm sure I can do it).***

|  | **0 = (I can't do it at all** | | **1** | **2** | **3** | **4** | **5 = I'm quite I can do it** | | | **6** | **7** | **8** | **9** | **10 = I'm sure I can do it** | | |
| --- | --- | --- | --- | --- | --- | --- | --- | --- | --- | --- | --- | --- | --- | --- | --- | --- |
| Break an upsetting problem down into smaller parts | O | O | | O | O | O | | O | O | | O | O | O | | O |  |
| Sort out what can be changed, and what cannot be changed | O | O | | O | O | O | | O | O | | O | O | O | | O |  |
| Make a plan of action and follow it when confronted with a problem | O | O | | O | O | O | | O | O | | O | O | O | | O |  |
| Leave options open when things get stressful | O | O | | O | O | O | | O | O | | O | O | O | | O |  |
| Think about one part of the problem at a time | O | O | | O | O | O | | O | O | | O | O | O | | O |  |
| Find solutions to your most difficult problems | O | O | | O | O | O | | O | O | | O | O | O | | O |  |
| Make unpleasant thoughts go away | O | O | | O | O | O | | O | O | | O | O | O | | O |  |
| Take your mind off unpleasant thoughts | O | O | | O | O | O | | O | O | | O | O | O | | O |  |
| Stop yourself from being upset by unpleasant thoughts | O | O | | O | O | O | | O | O | | O | O | O | | O |  |
| Keep from feeling sad | O | O | | O | O | O | | O | O | | O | O | O | | O |  |
| Get friends to help you with the things you need | O | O | | O | O | O | | O | O | | O | O | O | | O |  |
| Get emotional support from friends and family | O | O | | O | O | O | | O | O | | O | O | O | | O |  |
| Make new friends | O | O | | O | O | O | | O | O | | O | O | O | | O |  |

***Following, you’ll find 5 statements that describe how a person can feel when thinking about the risk of being infected from the new Coronavirus (COVID-19). Each sentence can be completed by choosing one of the 4 specific states, or the intermediate points between the different states. Please, indicate the position that best indicates your state, by clicking on the corresponding dot.***

***Please, check that you have answered all the statements and that you have indicated only one option for each of them.***

*.*

| *Thinking about the management of your illness in this emergency...* | | | | | | | |  |
| --- | --- | --- | --- | --- | --- | --- | --- | --- |
|  |  |  |  |  |  |  |  |  |
| *1* | I feel like I’m in blackout  O | O | I feel in alert  O | O | I feel informed  O | O | I feel positive  O |  |
| *2* | I feel lost  O | O | I feel alarmed  O | O | I feel conscious  O | O | I feel in peace  O |  |
| *3* | I feel overwhelmed by emotions  O | O | I feel in anxiety every time I hear talking about the Covid-19  O | O | I feel I got used to this emergency  O | O | My life goes on regardless of this situation  O |  |
| *4* | I'm living moments of great discouragement  O | O | I often feel anxious  O | O | I feel I got used to this situation  O | O | I am generally optimist about my future and my health  O |  |
| *5* | I feel paniked  O | O | I feel the urgency to do something  O | O | I keep calm  O | O | I am in control  O |  |

1. **Disease management during the Covid-19 health emergency**

***In addition to the daily management of your disease, the Covid-19 health emergency may have an impact on your relationship with your treatment system. Based on your experience, please answer the following questions.***

***Did you cancel hospital visits for a gastrointestinal check-up or treatment for fear of***

***contracting Covid-19?***

- - Yes
  - No

***Are you having trouble contacting your physician because of the Covid-19 emergency?***

- - Yes
  - No
  - I didn’t try to contact my doctor

***Are the patient associations of reference for my disease very helpful in supporting me in this period of emergency?***

- - Yes
  - No

***Can you stay in contact with patient associations despite the emergency?***

- - Yes
  - No

***Did you stop taking your medication because of the spread of Covid-19?***

- - Yes, independently
  - Yes, on advice of doctor
  - No
