# Supplementary material for: Psychological reaction to Covid-19 of Italian patients with IBD
Source: BMC Psychol. 2021 Aug 6;9:115. doi: 10.1186/s40359-021-00622-6 (PMC8343359; doi:10.1186/s40359-021-00622-6)
Supplement: Supplementary file 2 — Additional file 2. Graphs about Covid-19 general concerns. [file 40359_2021_622_MOESM2_ESM.docx]

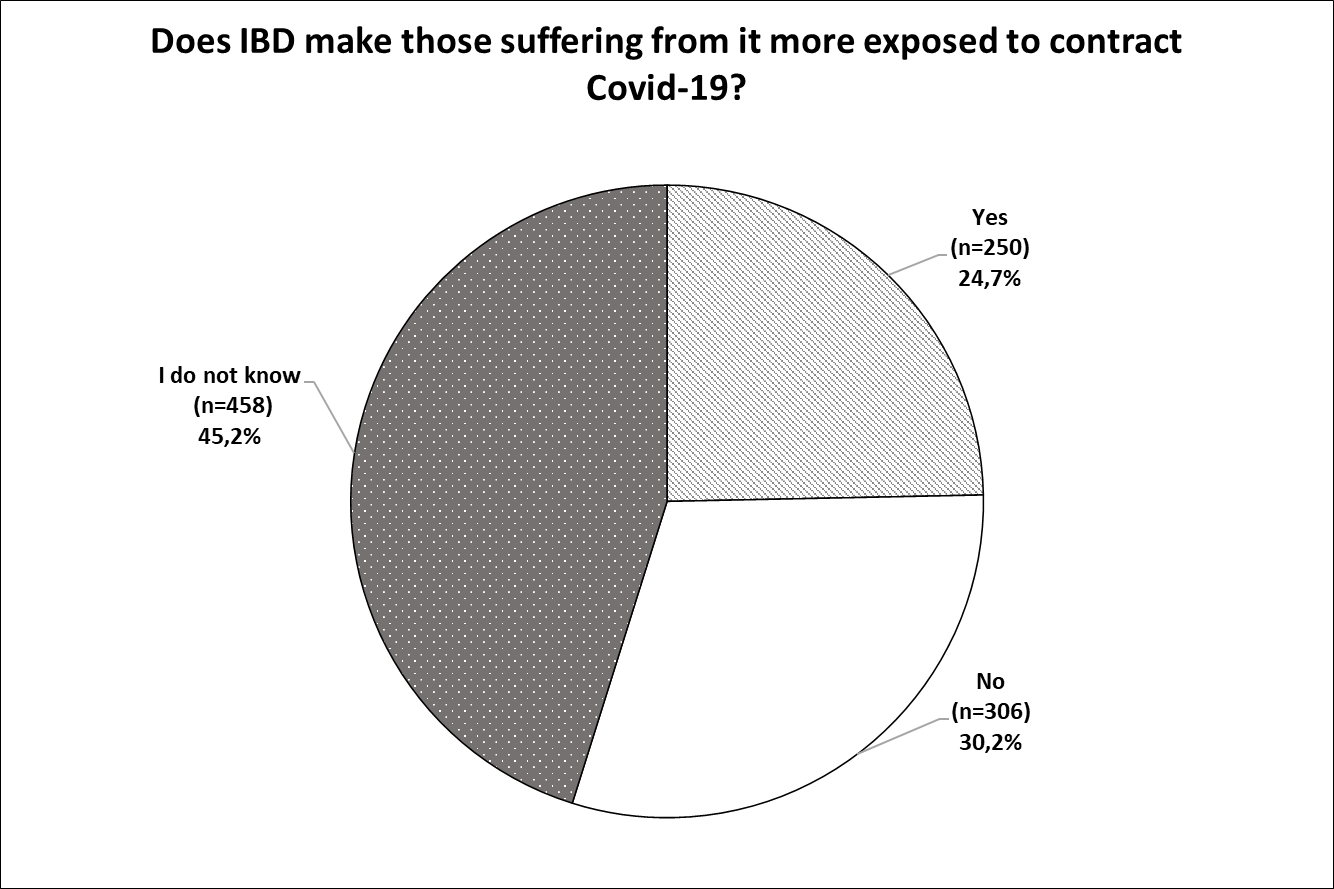


**Fig 1**. IBD and possibility to contract Covid-19 (N=1014)

**Fig 2**. IBD and possibility of worsening because of Covid-19 (N=1014)

**Fig 3**. Using immunosuppressants and exposure to contagion from Covid-19 (N=1014)

**
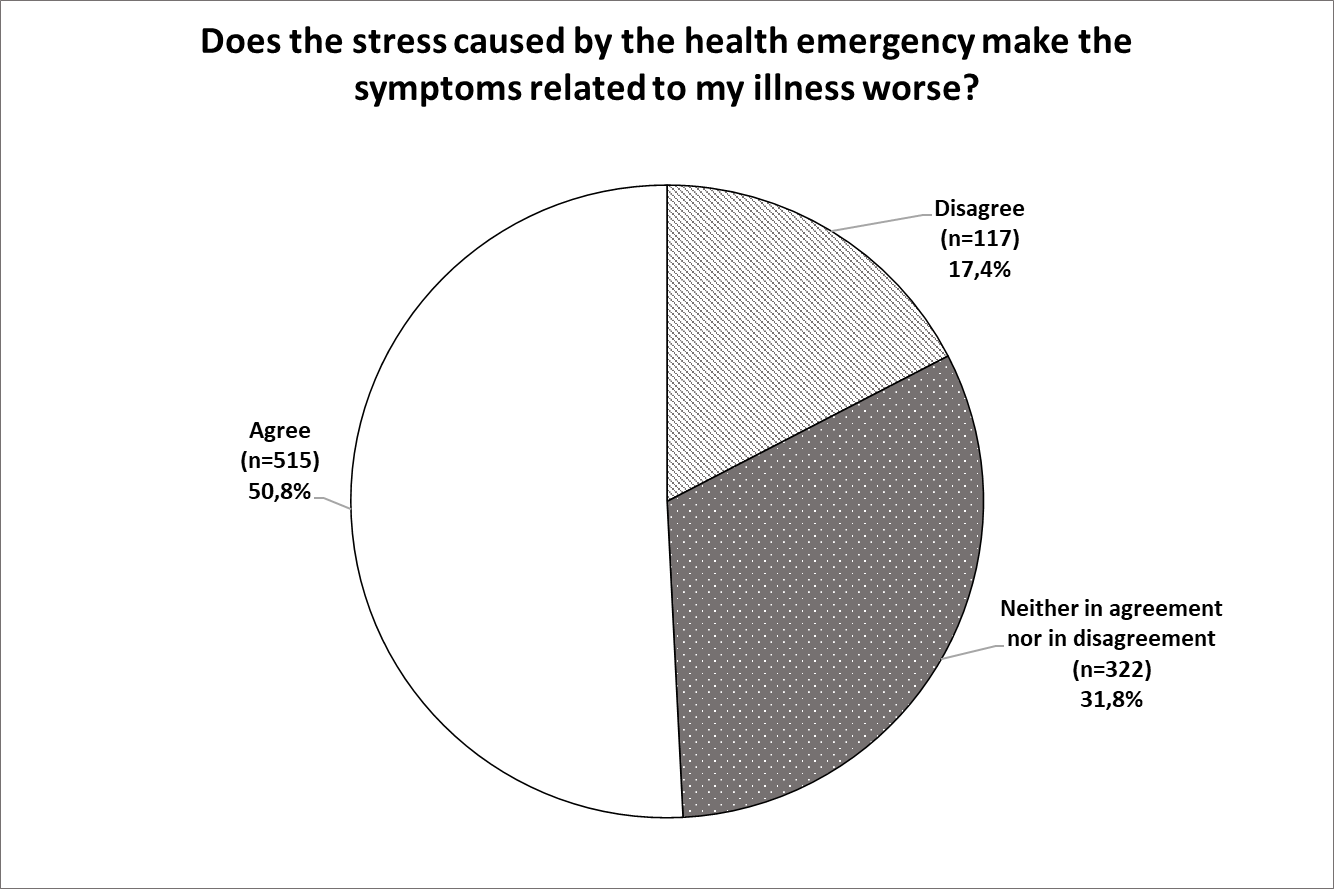
**

**Fig 4**. IBD and stress (N=1014)
