# Supplementary material for: Psychological reaction to Covid-19 of Italian patients with IBD
Source: BMC Psychol. 2021 Aug 6;9:115. doi: 10.1186/s40359-021-00622-6 (PMC8343359; doi:10.1186/s40359-021-00622-6)
Supplement: Supplementary file 3 — Additional file 3. Graphsabout Disease management during the Covid-19 health emergency. [file 40359_2021_622_MOESM3_ESM.docx]

**Fig 5**. Cancellation of hospital visits (N=1014)

**Fig 6**. Medicine interruption (N=1014)

**Fig 7**. Ability to keep in touch with patient associations (N=1014)

**Fig 8**. Usefulness of patient associations (N=1014)

**Fig 9**. Difficulty in contacting the referring doctor (N=1014)
